# Fibrous and Spherical Aggregates of Ovotransferrin as Stabilizers for Oleogel-Based Pickering Emulsions: Preparation, Characteristics and Curcumin Delivery

**DOI:** 10.3390/gels8080517

**Published:** 2022-08-19

**Authors:** Qi Zhou, Zihao Wei, Yanan Xu, Changhu Xue

**Affiliations:** 1 College of Food Science and Engineering, Ocean University of China, Qingdao 266003, China; 2 Qingdao National Laboratory for Marine Science and Technology, Qingdao 266235, China

**Keywords:** oleogel-based Pickering emulsions, aggregates, ovotransferrin fibrils, ovotransferrin spheres, medium-chain triglyceride oil, nutrient delivery

## Abstract

This study aimed to explore the effects and mechanisms of differently shaped aggregates of ovotransferrin (OVT) particles on oleogel-based Pickering emulsions (OPEs). Medium-chain triglyceride oil-based oleogels were constructed using beeswax, and their gel-sol melting temperatures were investigated. Atomic force microscopy confirmed that both OVT fibrils and OVT spheres were successfully prepared, and the three-phase contact angle measurements indicated that fibrous and spherical aggregates of OVT particles possessed great potential to stabilize the OPEs. Afterward, the oil-in-water OPEs were fabricated using oleogel as the oil phase and OVT fibrils/spheres as the emulsifiers. The results revealed that OPEs stabilized with OVT fibrils (FIB-OPEs) presented a higher degree of emulsification, smaller droplet size, better physical stability and stronger apparent viscosity compared with OPEs stabilized with OVT spheres (SPH-OPEs). The freeze–thaw stability test showed that the FIB-OPEs remained stable after three freeze–thaw cycles, while the SPH-OPEs could barely withstand one freeze–thaw cycle. An in vitro digestion study suggested that OVT fibrils conferred distinctly higher lipolysis (46.0%) and bioaccessibility (62.8%) of curcumin to OPEs.

## 1. Introduction

Oleogel is manufactured when the gelator structures a thermally reversible three-dimensional gel network in the flowing oil via Van der Waals forces and hydrogen interactions. At present, oleogel has attracted much attention due to its structural diversity, various gelling mechanisms, convenient processing and easy affordability [1]. In the field of food science, oleogel plays an important role in improving the solubility and physical stability of certain nutrients (DHA, resveratrol, β-carotene, etc.), inhibiting oxidation and achieving the sustained release effect [2,3,4]. Currently, a series of studies demonstrated that the oleogel structure improved the stability and bioavailability of the delivered bioactive compounds (such as curcumin, quercetin, hesperidin, etc.) [5,6]. A recent study indicated that oleogel could also protect lutein ester from UV radiation [7]. Furthermore, another study showed that the loaded nutrients did not interfere with the assembly of the oleogel structure [8]. At the same time, these systems can be used as substitutes for saturated fats and hydrogenated oils in a wide range of applications in confectionery products, meat products and various supplements, presenting satisfactory function, texture, color and taste [9,10,11,12]. 

Oleogel can be further emulsified to obtain oleogel-based emulsion, which provides a relatively novel approach for avoiding the high viscosity and hydrophobicity of oleogels, improving the versatility of the designed systems and displaying great potential to be widely applied in the food industry. Several studies on the preparation and characterization of oleogel-based emulsions have been published [13,14,15]. The results indicated that oleogel-based emulsions possessed a good storage stability, satisfactory particle size and excellent oil binding capacity (>90%). Moreover, a recent study has shown that oleogel-based emulsions also have great potential in capturing probiotics and improving their activity while preventing lipid oxidation [16]. In particular, edible oleogel-based Pickering emulsions (OPEs) have received considerable attention owing to the absence of unhealthy small-molecular surfactants and outstanding stability [17]. It is worth mentioning that the structure composed of the oleogel acting as an internal phase of OPEs and the emulsifiers adsorbed at the oil–water interface can also be related to nanostructured lipid carriers (with a different particle size) [1], which indicates that OPEs can also exert the advantages of high loading capacity and strong stability of nanostructured lipid carriers. In a previous study, OPEs were constructed and showed good storage stability [18]. The in vitro digestion study by Xia et al. revealed that OPEs were able to significantly increase the rate of lipolysis and the bioaccessibility of the delivered nutrient [19]. 

Protein particles have recently been increasingly used as solid-particle stabilizers of OPEs because of their generally considered safety, unique interfacial properties and wide sources [20,21,22]. Ovotransferrin (OVT), composed of 686 amino acids, is a common glycoprotein in egg white. Its excellent properties, including iron supplement, antibacterial, anti-inflammatory and regeneration, have gained increasing appreciation [17,23]. Under specific processing conditions, OVT particles have the ability to self-assemble into different shapes of aggregates at the supramolecular level. Thereinto, thermal denaturation under suitable conditions (for example, far from the isoelectric point) may cause protein particles to aggregate into fibrils. The fibrils obtained tend to have high aspect ratio anisotropy, and they may possess special interfacial behavior under capillary forces [24]. Furthermore, previous studies indicated that OVT particles presented great potential to form spherical aggregates with excellent irreversible interfacial adsorption [25,26,27]. It can be inferred that OVT spheres are able to adsorb at the oil/water interface to form a film that protects the emulsion droplets from flocculation and coalescence. However, the effects and mechanisms of fibrous and spherical aggregates on OPEs are still unclear at present, which hinders the selection of different shapes of aggregates to construct the food system, the control of conditions during food production and the acquisition of products with satisfactory properties. Therefore, exploring the effects and mechanisms of fibrous and spherical aggregates on OPEs is of great significance to determine the most suitable production conditions to reduce production cost and obtain products that satisfy people’s demands for nutrition and health. 

Accordingly, the objectives of this study were to verify the feasibility of stabilizing OPEs with OVT fibrils/spheres, to characterize the OPEs stabilized with OVT fibrils (FIB-OPEs) and the OPEs stabilized with OVT spheres (SPH-OPEs), and to explore the effects and mechanisms of different shapes of OVT particle aggregates on OPEs for the first time. Firstly, OVT fibrils and OVT spheres were prepared, and then, their microstructure and three-phase contact angles were measured. Afterward, oleogel was prepared, acting as the internal oil phase of OPEs, and the OVT spheres/fibrils were used as particle stabilizers to prepare the OPEs. The type, creaming index, droplet size, physical stability, rheological properties and freeze–thaw stability of FIB-OPEs and SPH-OPEs were investigated. Finally, the bioaccessibility of encapsulated curcumin as well as the lipolysis of FIB-OPEs and SPH-OPEs were explored. This study may provide guidance for finding the most suitable stabilizers of OPEs to gain the required characteristics, determining suitable production conditions and producing products that can satisfy various human requirements.

## 2. Results and Discussion

### 2.1. Preparation and Characterization of BW-MCT Oleogels

Beeswax (BW), with satisfactory melting point, hardness, viscosity and good gel properties, is widely applied in food and pharmaceutical fields [28]. Recent research works have shown that medium-chain triglycerides can inhibit visceral fat accumulation while providing a rapid energy source for human body to replace sugar, thus having great potential in the fight against obesity, cardiovascular disease and diabetes caused by excessive consumption of dietary oils and fats [29]. Therefore, BW as the gelator and medium-chain triglyceride oil (MCT) as the base oil were chosen to fabricate oleogels. The three-dimensional reticular structure formed by the crystallization and coalescence of lipid in beeswax restricted the movement of liquid oil, and the acquired oleogels exhibited excellent adhesion and cohesion [30]. As shown in Table 1, the BW concentration required for the formation of oleogel was at least 1.6% (*w*/*v*). 

Thermoreversible oleogels can be converted into the sol state at higher temperatures and re-gelled at lower temperatures [31], which has significant implications for food processing and storage, and therefore, the gel-sol melting temperatures of BW-MCT oleogels were investigated (Table 1). With the increase in the amount of BW, the intermolecular hydrogen bond increased, and thus, the melting temperature of the oleogel rose. As shown in Table 1, the oleogel structured with 1.6% (*w*/*v*) BW had the lowest T_m_ (32.5 °C) among all investigated BW-MCT oleogels. Hence, it is important to note that the oleogel systems containing 1.6% (*w*/*v*) BW involved in this study are suitable for room temperature storage or refrigeration. It was obvious that oleogels in the sol state could adsorb more emulsifiers due to their larger specific surface area. Accordingly, the oleogel with low gel-sol melting temperature could turn to sol state with the help of the friction heat generated (which increased the temperature of the system from 26.5 °C to 33.0 °C) by high-speed shearing during the preparation of the emulsions, which indicated that the BW-MCT oleogel with low BW content had great potential to be used as the internal oil phase to adsorb the emulsifiers and form emulsions. When the system temperature returned to room temperature, the sol state turned to the gel state again. As a result, the oleogel with 1.6% (*w*/*v*) BW addition was more inclined to successfully constructed OPEs at a relatively low temperature. In consideration of easier OPEs formation as well as the lower product cost, the oleogel structured with 1.6% (*w*/*v*) BW was used in the subsequent parts of this study. 

### 2.2. Formation and Characterization of OVT Fibrils and OVT Spheres

During the formation of OVT fibrils, the critical aggregation concentration of protein reduced distinctly with the increase in ionic strength. Moreover, in the growth stage of fibrillation, moderate ionic strength was conducive to the formation of OVT fibrils. Based on the above considerations, the ionic strength of 150 mM was selected for the preparation of OVT fibrils in this study. The high temperature (90 °C) chosen in this study was more suitable for fiber formation due to the enhanced hydrophobic interactions and the increased frequency of collisions of molecules at higher temperatures [32]. Previous studies have shown that OVT fibrils cannot be formed at pH values relatively close to the isoelectric point [33]. In addition, taking the impact of pH value on product taste into consideration, the pH 2.0 was selected for fibrillation. In terms of the preparation of OVT spheres, higher salt concentration (1 M) could adjust the structure and morphology of the aggregate through the screening effect, which contributed to the formation of spherical aggregates [34]. On the other hand, when the pH (7.5) was close to the isoelectric point (6.2) of OVT, the net charge and electrostatic repulsion of OVT were minimal [33], resulting in the generation of spherical aggregates.

**Figure 1:**
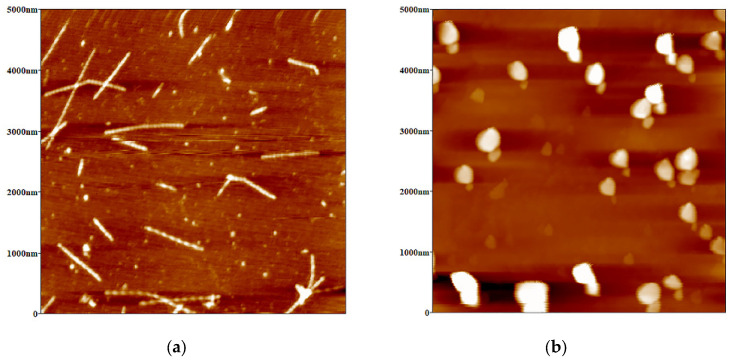
(**a**) Atomic force microscopy (AFM) images of ovotransferrin (OVT) fibrils; (**b**) AFM images of OVT spheres.

The microstructure of OVT spheres and OVT fibrils was characterized by atomic force microscopy (AFM). Figure 1 confirmed that rod-like fibrils with relatively uniform length and thickness as well as OVT spheres of similar diameter were formed. According to the Nano Scope Analysis Software, the average length of the OVT fibrils and the diameter of the OVT spheres were 620 nm and 395 nm, respectively. It was apparent that the droplet size of Pickering emulsions could be several microns [35], which implied that both the obtained fibrils and spheres had great potential for the stabilization of OPEs because the size of the eligible emulsifier should be much smaller than the droplet size of the target emulsions.

**Figure 2:**
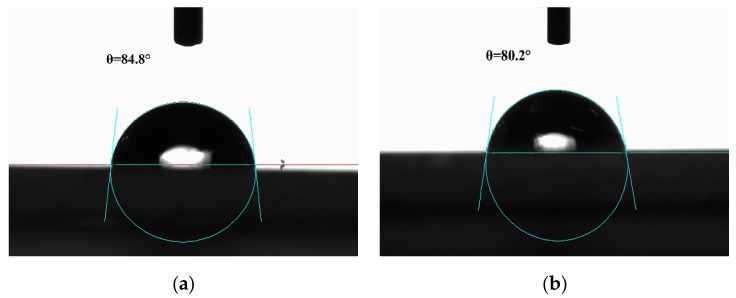
(**a**) Three-phase contact angle (oil–water-solid) of OVT fibrils; (**b**) Three-phase contact angle (oil–water-solid) of OVT spheres.

A prerequisite for a qualified stabilizer of Pickering emulsion systems is the partial wettability in both oil and water phases. The wettability of OVT fibrils/spheres in oil and water phases was reflected by a three-phase contact angle (oil–water-solid) to verify if it was a qualified stabilizer of OPEs [36]. Figure 2 shows that the three-phase contact angles of OVT fibrils and OVT spheres were 84.8° and 80.2°, respectively. Generally, solid particles are considered hydrophilic when θ < 90° and hydrophobic when θ > 90° [37]. When the contact angle is about 90°, the hydrophilicity of the particles is slightly stronger or weaker than hydrophobicity, and the difference between the energy needed to transfer particles from the interface to the single phase and the energy needed to transfer particles from the single phase to the interface is small. As a result, the solid particles can be adsorbed at the oil–water interface, and thus, the Pickering emulsion formed possesses good stability. Therefore, it could be inferred that both OVT fibrils and OVT spheres were able to be partially wetted by water and oil simultaneously, indicating that these two aggregates with different shapes had good adsorption at the oil–water interface and could satisfactorily perform as stabilizers of OPEs. Furthermore, compared with OVT spheres, OVT fibrils were more hydrophobic. This may be because the acid heat treatment altered the molecular structure of OVT and exposed internal hydrophobic regions during fibril formation, thereby weakening the hydrophilic characteristics.

### 2.3. Preparation of FIB-OPEs and SPH-OPEs

**Figure 3:**
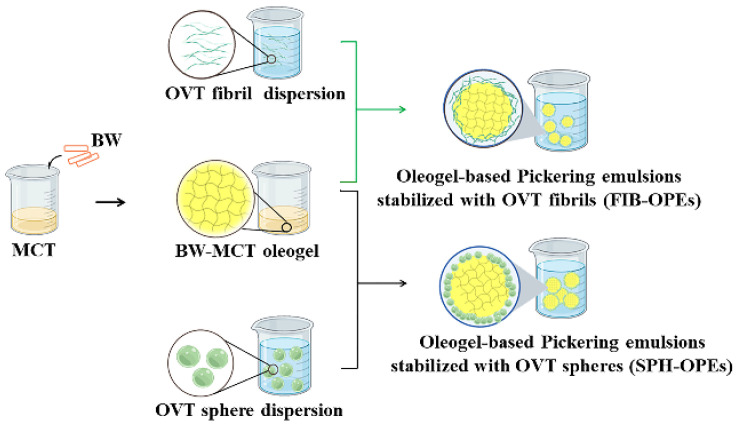
Schematic diagram of the preparation process of oleogel-based Pickering emulsions stabilized with OVT fibrils (FIB-OPEs) and oleogel-based Pickering emulsions stabilized with OVT spheres (SPH-OPEs).

**Figure 4:**
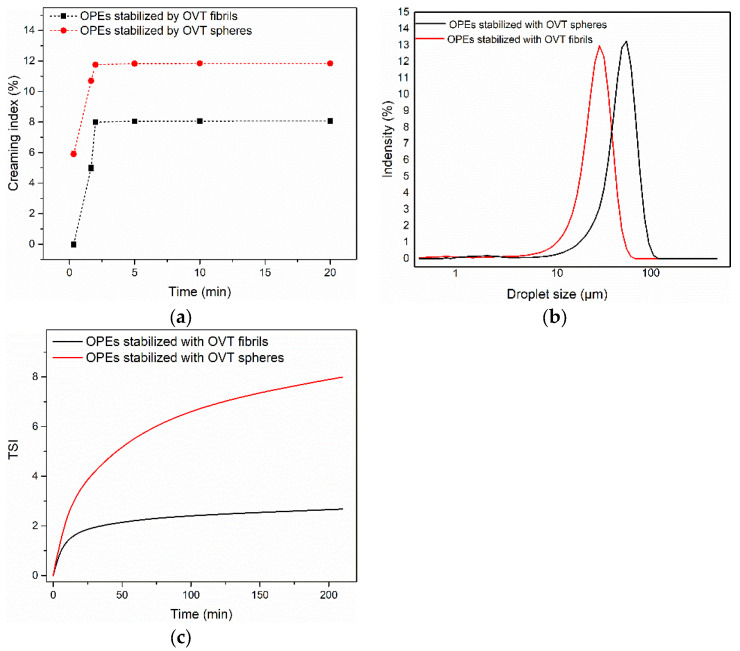
(**a**) The creaming index of FIB-OPEs and SPH-OPEs; (**b**) Average droplet size of FIB-OPEs and SPH-OPEs; (**c**) TSI profile of FIB-OPEs and SPH-OPEs.

OPEs were prepared with BW-MCT oleogel as the oil phase and OVT fibrils/spheres as stabilizers (Figure 3). Because all of the investigated OPEs dispersed instantly in water and remained insoluble in MCT, FIB-OPEs and SPH-OPEs were determined as oil-in-water emulsions [19]. A confocal laser scanning microscopy (CLSM) was performed to visualize the microstructure of FIB-OPEs and SPH-OPEs. The green filled circles and the red circles corresponding to MCT and OVT fibrils/spheres can be clearly identified in Figure S1a,b and Figure S1d,e. As shown in Figure S1c and Figure S1f, the green filled circle was surrounded by the red circle, verifying that the oil-in-water FIB-OPEs and SPH-OPEs were successfully fabricated. Furthermore, the CLSM observation also revealed that the droplets of obtained FIB-OPEs and SPH-OPEs were regular spherical. At the OVT fibril/sphere concentration of 40 mg/mL and oleogel fraction of 0.50, the creaming index of the freshly prepared OPEs was determined. Figure 4a reflects the trends of the emulsion phase and serum phase of the samples. After 200 s, the creaming index of FIB-OPEs remained rather constant around 8%, while the creaming index of SPH-OPEs remained fairly stable around 12%, indicating that both FIB-OPEs and SPH-OPEs presented good stability. The favorable stability could be attributed to the high energy barrier due to the irreversible adsorption of OVT spheres/fibrils at the oil–water interface [38]. It is worth noting that the BW-MCT oleogel used as the oil phase of emulsions also played a positive role in the satisfactory stability of OPEs. Notably, more creaming was observed in SPH-OPEs than FIB-OPEs. Moreover, SPH-OPEs exhibited a rapid growth rate of creaming in comparison to FIB-OPEs. Therefore, it could be considered that FIB-OPEs displayed better stability. The reason may be that, as anisotropic aggregates, OVT fibrils presented better emulsifying effects than OVT spheres owing to a larger aspect ratio [23].

### 2.4. Droplet Size

In view of the significant impact of droplet size on the characteristics of OPEs, the droplet size of FIB-OPEs and SPH-OPEs was measured. Figure 4b depicts the droplet size of the prepared FIB-OPEs and SPH-OPEs. The single peak indicated that both FIB-OPEs and SPH-OPEs with uniform particle sizes were successfully formed. The droplet size of FIB-OPEs was mainly distributed in 60.04–67.55 μm, and the droplet size of SPH-OPEs was mainly distributed in 67.55–75.98 μm. The droplet size of SPH-OPEs was larger than that of FIB-OPEs, which was consistent with the results of creaming index in Section 2.3 because the creaming rate was proportional to the square of droplet radius according to Stokes’ law. The bigger droplet size of SPH-OPEs could be expounded by the following reasons. Compared to OVT fibrils, the smaller size and relatively weaker lipophilicity of the OVT spheres resulted in their less dense coverage on the oil droplets than the fibrils, which may lead to coalescence of the oil droplets, resulting in larger droplet size of the emulsions. Additionally, OVT fibrils with the average length larger than OVT spheres required more energy to separate from the oil–water interface, which also contributed to avoiding droplet aggregation and obtaining independent droplets with small size.

### 2.5. Physical Stability

To investigate the physical stability of FIB-OPEs and SPH-OPEs, the TSI obtained by Turbiscan LAB was employed to evaluate the migration state of emulsions. The physical stability of OPEs was negatively correlated with the TSI value [39]. As illustrated in Figure 4c, the migration rate of droplets in both FIB-OPEs and SPH-OPEs decreased with the passage of time, and FIB-OPEs tended to present good stability after 3.5 h. Ultimately, the TSI value of FIB-OPEs was significantly lower than that of the SPH-OPEs, indicating that fibrils possessed a higher potential to stabilize the Pickering emulsion systems. This was because flexible OVT fibrils were easier for changing the conformation compared with OVT spheres [40]. The flexibility and conformational changes of fibrils contributed to the absorption on oil–water surfaces [41], which was consistent with the wettability results in Section 2.2. In this regard, using OVT fibrils to stabilize OPEs had better barrier properties and lower permeability, and the obtained OPEs were less liable to coacervation, showing good physical stability.

### 2.6. Freeze–Thaw Stability

**Figure 5:**
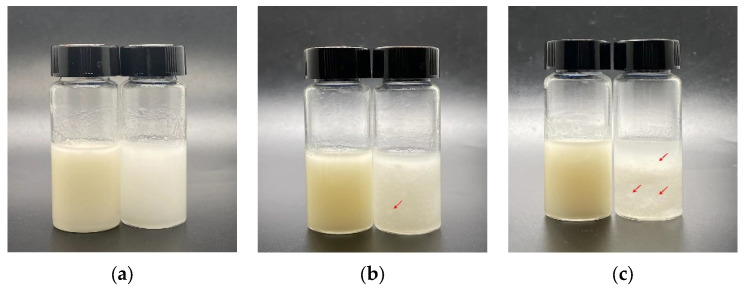
Photograph of FIB-OPEs (left) and SPH-OPEs (right): (**a**) Visual observation of initial OPEs; (**b**) Visual observation of OPEs after one freeze–thaw cycle; (**c**) Visual observation of OPEs after three freeze–thaw cycles. There was oil droplet precipitation at the position marked with the red arrow.

A number of foods are stored temporarily by freezing and heated to thaw before eating, and thus, it is of great importance to investigate the freeze–thaw stability of FIB-OPEs and SPH-OPEs. Figure 5a shows that freshly prepared OPEs were homogeneous and almost only involved the emulsified phase. After undergoing one freeze–thaw cycle, SPH-OPEs began to have a little oil precipitation, while FIB-OPEs remained homogeneous and stable (Figure 5b). Considering that emulsion products could be regarded as stable when the overall appearance has no significantly visible change [42], the samples were considered to have the ability to undergo one freeze–thaw cycle. When completing three freeze–thaw cycles, significant oiling-off was observed in SPH-OPEs, and the emulsified phase volume decreased (Figure 5c), suggesting that SPH-OPEs exhibited poor freeze–thaw stability. On the contrary, FIB-OPEs presented consistent stability without obvious oil–water separation (Figure 5c). The FIB-OPEs showed better freeze–thaw stability than the SPH-OPEs, which may be due to the formation of large amounts of ice crystals penetrating into the oil droplets and thereby disrupting the interfacial emulsifier layers composed of OVT spheres [43]. In the case of FIB-OPEs, the fibrils possessed longer contour length and excellent flexibility, which inhibited the destruction of the interfacial emulsifier layers by ice crystals.

### 2.7. Rheological Analysis

**Figure 6:**
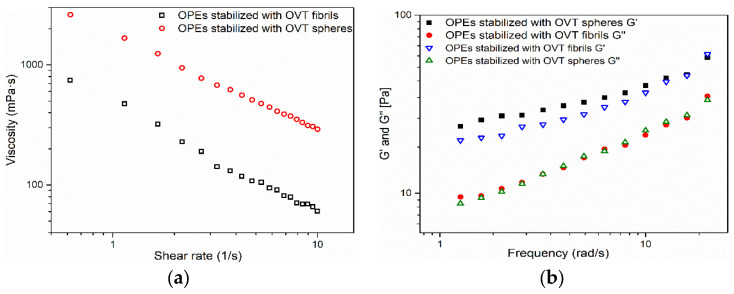
(**a**) Apparent viscosity of FIB-OPEs and SPH-OPEs at 25 ± 0.1 °C; (**b**) Storage modulus (G′) and loss modulus (G″) of FIB-OPEs and SPH-OPEs at 25 ± 0.1 °C.

Rheology is closely linked with food processing, appearance, texture, product taste and shelf life [44]. Figure 6 shows the apparent viscosity, loss modulus (G″) and storage modulus (G′) of SPH-OPEs and FIB-OPEs. Since the BW-MCT oleogel was the internal oil phase of O/W OPEs, the three-dimensional network structure in OPEs was relatively loose, and the molecular interactions were weak under the encapsulation and dilution of the aqueous phase. As a result, FIB-OPEs and SPH-OPEs presented lower viscosity than the oleogel [45], which contributed to expanding the application in food (such as functional beverages). It can be seen from Figure 6a that the viscosity of FIB-OPEs (743 mPa·s at shear rate of 0.1 s^−1^) was lower than that of SPH-OPEs (2050 mPa·s at shear rate of 0.1 s^−1^). This was due to the extension of the flexible structure of OVT fibrils during the preparation process, and the van der Waals forces, electrostatic bonds as well as hydrophobic interactions were disrupted [46]. Additionally, both FIB-OPEs and SPH-OPEs exhibited shear-thinning behavior. On the one hand, the rearrangement of the OPEs droplets in the flow direction reduced the flow resistance, thus resulting in the shear-thinning properties. On the other hand, the possible destruction of the internal gel structure under a high shear rate may also lead to the shear-thinning behavior of OPEs. In Figure 6b, G′ represents the energy stored in the fluid because of elastic deformation, whereas G″ represents the energy lost because of viscous deformation. The G′ of SPH-OPEs was slightly higher than that of FIB-OPEs, which could be explained by the highly elastic film formed by rigid OVT spheres at the O/W interface [23]. It is worth noting that the G″ of SPH-OPEs and FIB-OPEs was roughly the same in the frequency range of 0.1–20 rad/s, denoting that there was no obvious difference in lost deformation energy between these two emulsions.

### 2.8. The Light Stability of Curcumin Encapsulated in FIB-OPEs and SPH-OPEs

**Figure 7:**
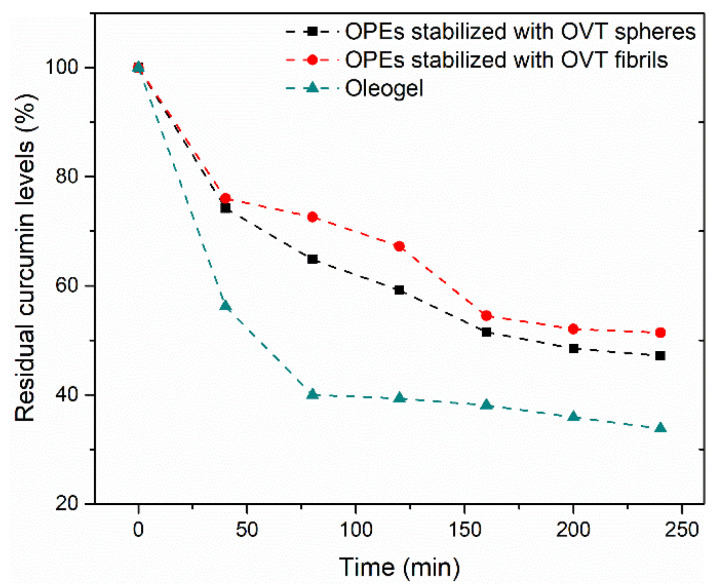
Residual curcumin levels in oleogel, FIB-OPEs and SPH-OPEs under natural light treatment.

Curcumin exhibits excellent bioactivity, including antioxidant activity, anti-inflammatory activity and immune-regulatory activity [47]. However, it is sensitive to light, which prevents its wide application in food. Therefore, the protective effects of FIB-OPEs and SPH-OPEs on the delivered curcumin under natural light were explored. The BW-MCT oleogel loaded with curcumin was tested as the control. As depicted in Figure 7, curcumin encapsulated in oleogel was degraded rapidly in comparison to that in FIB-OPEs and SPH-OPEs, which indicated that the FIB-OPEs and SPH-OPEs provided an effective strategy to protect curcumin under natural light. This was because the curcumin in the oleogel was readily exposed to natural light, resulting in the absorption of more energy and accelerated degradation, whereas the OVT spheres and OVT fibrils in the FIB-OPEs and SPH-OPEs adsorbed at the oil–water interface and formed a barrier, shielding curcumin from natural light and delaying energy transmission. After 4 h of natural light treatment, the residual percentages of curcumin in FIB-OPEs and SPH-OPEs were 51.42% and 47.16%, respectively, which was higher than those in the protein–polysaccharide–surfactant ternary complexes constructed by Guo et al. [48]. Notably, the residual percentage of curcumin in FIB-OPEs was higher than that in SPH-OPEs, which may be related to the degree of adsorption of OVT spheres/fibrils at the oil–water interface and the compactness of the formed interfacial film.

### 2.9. Lipolysis and Bioaccessibility of Curcumin Encapsulated in FIB-OPEs and SPH-OPEs

**Figure 8:**
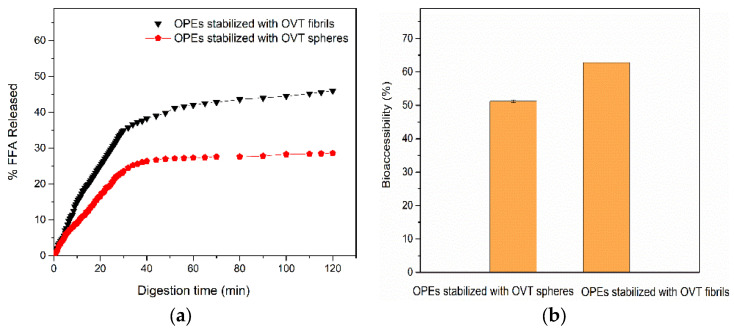
(**a**) Release profile of free fatty acids (FFA) in FIB-OPEs and SPH-OPEs; (**b**) The bioaccessibility of curcumin encapsulated in FIB-OPEs and SPH-OPEs.

A lipolysis analysis was performed to evaluate the digestibility of lipid in FIB-OPEs and SPH-OPEs during in vitro digestion [49]. The lipolysis result in Figure 8a was based on the assumption that digesting 1 mol of triglyceride consumed 2 mol of free fatty acid (FFA). SPH-OPEs (28.6%) and FIB-OPEs (46.0%) had a higher FFA released fraction than the MCT-based emulsions (16.0%) and OPEs stabilized with ovotransferrin–carboxymethyl chitosan nanoparticles (23.0%) [18,50], which presented the favorable delivery properties of SPH-OPEs and FIB-OPEs. As shown in Figure 8a, the FFA released fraction of SPH-OPEs (28.6%) was lower than that of FIB-OPEs (46.0%), indicating that FIB-OPEs had a higher extent of lipolysis. The higher extent of lipolysis in FIB-OPEs may be accounted for by the following factors. Firstly, the specific surface area of fibrils was larger than that of spheres, which contributed to higher pepsin exposure, and the interfacial fibril film was destroyed more easily, resulting in more oleogel being released from the inner phase for subsequent digestion. Additionally, the droplet size of FIB-OPEs was smaller than that of SPH-OPEs, which facilitated the adsorption of lipase at the lipase–oil interface and accelerated lipolysis. 

The gastrointestinal fate of the encapsulated curcumin in the emulsions was investigated from the perspective of in vitro bioaccessibility to analyze and compare the loading characteristics of SPH-OPEs and FIB-OPEs. As shown in Figure 8b, the bioavailability of curcumin in FIB-OPEs and SPH-OPEs was 62.8% and 51.2%, respectively, while that of curcumin in oleogel was ≤47% in previous studies [51,52]. The higher bioavailability may be due to the better dispersibility and smaller size of oleogels in the FIB-OPEs and SPH-OPEs that were more fully digested than oleogels alone, allowing more curcumin to be released. Moreover, the bioaccessibility of curcumin encapsulated in FIB-OPEs/SPH-OPEs was significantly higher than that of curcumin in organogel-based nanoemulsions (around 40%) constructed in a previous study, demonstrating the satisfactory delivery and protection properties of SPH-OPEs and FIB-OPEs for curcumin [51]. In particular, the bioaccessibility of curcumin in FIB-OPEs was higher than that in SPH-OPEs, which may be related to the release of more FFA from FIB-OPEs. Previous studies have shown that curcumin is stored in micelles before being absorbed by small intestinal epithelial cells [53,54]. More micelles containing released curcumin were generated after simulated gastrointestinal digestion, which suggested more bioaccessible curcumin encapsulated in FIB-OPEs could be acquired in the human body. Accordingly, curcumin can be delivered more effectively via FIB-OPEs.

## 3. Conclusions

In conclusion, the physicochemical characteristics, as well as delivery properties of FIB-OPEs and SPH-OPEs, were analyzed, and the effects and mechanisms of different shapes of OVT particle aggregates on OPEs were explored in this study. Both OVT spheres and OVT fibrils could be used as emulsifiers to effectively stabilize OPEs. The results revealed that FIB-OPEs possessed a smaller droplet size, lower creaming index and better physical stability. In comparison with SPH-OPEs, OVT fibrils endowed OPEs with higher freeze–thaw stability, allowing FIB-OPEs to undergo at least three freeze–thaw cycles, whereas SPH-OPEs could barely withstand one freeze–thaw cycle. In terms of the delivery capacity, the FIB-OPEs presented significantly higher bioaccessibility of curcumin and lipolysis. The results obtained in this study are conducive to the selection and development of emulsifiers for OPEs and provide a basis for the food industry to determine the most appropriate production conditions and produce products with the required characteristics.

## 4. Materials and Methods

### 4.1. Materials

Beeswax (BW, purity > 90.0%, density 0.97 g/mL at 20 °C, melting point 62.0–67.0 °C) and medium-chain triacylglycerol oil (MCT, density 0.95 g/mL at 20 °C, the content of medium-chain triacylglycerol ≥ 99.5%) were purchased from Yuanye Biological Co., Ltd. (Shanghai, China) and Guangzhou Yongsheng Industry & Trade Co., Ltd. (Guangzhou, China), respectively. Ovotransferrin (OVT, protein content > 87%) was acquired from Neova Technologies Inc. (Abbotsford, Canada). All other chemical reagents were provided by Sinopharm Chemical Reagent Co., Ltd. (Shanghai, China). 

### 4.2. Preparation of BW-MCT Oleogel

The BW-MCT oleogel was prepared with BW as the gelator and MCT as the base oil. An appropriate amount of BW was added into the MCT, and the BW-MCT mixture in the vials was heated and stirred in an oil bath at 80 °C and 600 rpm until the BW was completely dissolved. Afterward, the homogeneous mixture in the vials was taken out and cooled to room temperature. When the mixture was completely cooled to room temperature (approximately 30 min), the vial was inverted, and the BW-MCT oleogel was considered to be successfully formed if it did not flow after 30 min of inversion.

### 4.3. Gel-Sol Melting Temperatures of BW-MCT Oleogel

The gel-sol melting temperatures of BW-MCT oleogels were measured through the typical tube inversion method [55]. BW-MCT oleogel samples structured by different amounts of BW were inverted and submerged below the water surface. The water temperature was set to gradually rise from room temperature at a rate of 0.5 °C/min. The samples in the vials were held for 1 min at each temperature point, and the flow/non-flow state was observed. The gel-sol melting temperature was the temperature at which the BW-MCT oleogel sample melted and flowed.

### 4.4. Preparation of OVT Fibrils

According to Wei et al. [32], OVT was distributed in Milli-Q water with pH 2.0 and ionic strength of 150 mM NaCl, followed by stirring at a low speed (350 rpm) to obtain a high-concentration OVT solution (65 mg/mL). The solution obtained was meticulously filtered through syringe filters (0.45 μm) and subsequently heated and stirred for 24 h via placement in an oil bath at 90 °C, 350 rpm. Afterward, the acquired OVT fibril dispersion was taken out and cooled rapidly. In order to remove NaCl, the OVT fibril dispersion was dialyzed.

### 4.5. Preparation of OVT Spheres

The pH, ionic strength and rotational speed were controlled to make OVT particles form spherical aggregates at the supramolecular level, and the other conditions were the same as those in Section 4.4. Briefly, OVT was placed in Milli-Q water with pH 7.5 and ionic strength of 1 M NaCl, followed by stirring at 500 rpm. The solution obtained was filtered and subsequently heated and stirred in an oil bath at 90 °C, 500 rpm for 24 h. Afterward, the acquired OVT fibril dispersion was taken out and cooled rapidly. In order to remove ions, the sphere dispersion was dialyzed.

### 4.6. Micromorphology

The micromorphology of OVT fibrils and OVT spheres was characterized by the tapping mode of the atomic force microscopy (AFM, Being Nano-Instruments Co., Ltd., Guangzhou, China). Considering that the micromorphology of OVT fibrils/spheres was not affected by the concentration [17], the prepared OVT fibril/sphere dispersion was diluted to 1 mg/mL to facilitate imaging and analyzing the micromorphology of OVT fibrils and OVT spheres. The sample solution (10 μL) was spread onto the fresh mica surface and then dried with a nitrogen stream. Nano Scope Analysis Software (Being Nano-Instruments Co., Ltd., Guangzhou, China) was used for image analysis.

### 4.7. Three-Phase Contact Angle Test

The partial wettability of OVT fibrils and OVT spheres was reflected by testing their three-phase contact angles. The OVT fibril and sphere dispersions were freeze dried, and then, the obtained powder was pressed into thin cylindrical tablets with a diameter of 10 mm and a thickness of 2 mm by a tablet machine (10 MPa, 2 min). Afterward, the obtained tablets were, respectively, placed in a cube optical glass cell, followed by adding MCT into the cell. Milli-Q water droplets (3 μL) were injected onto the upper surface of the tablets by the sessile drop method from a syringe, and then, the three-phase contact angles were tested by an OCA 15 EC contact angle measuring instrument (Dataphysics, Filderstadt, Germany) at room temperature.

### 4.8. Preparation of FIB-OPEs and SPH-OPEs

In consideration of the lower product cost and easier OPEs formation, the oleogel structured with 1.6% (*w*/*v*) BW was used as the internal oil phase of OPEs in subsequent studies. OVT fibril (diluted to 40 mg/mL) and OVT sphere (diluted to 40 mg/mL) dispersions were mixed with BW-MCT oleogel (1:1 by volume) at room temperature, respectively. The mixture of OVT fibrils/spheres and BW-MCT oleogel was subsequently homogenized using an Ultra-Turrax T25 (IKA-Werke GMBH & CO., Staufen, Germany) at 10,000 rpm for 4 min.

### 4.9. Emulsion Type

Once the emulsion was formed, the drop dilution test was applied to identify the type of OPEs. Specifically, the OPEs were dripped into water and MCT, respectively. The emulsion type was identified as an oil-in-water emulsion if it dispersed instantly in water and remained insoluble in MCT. Otherwise, the type of the prepared emulsion was a water-in-oil emulsion.

### 4.10. CLSM

An A1R HD25 CLSM (Nikon, Tokyo, Japan) in the fluorescence mode was performed to visualize the microstructure of FIB-OPEs and SPH-OPEs by localizing the oil droplets and OVT particles in the emulsions. Nile red solution in methanol (15 μL, 0.01% *w*/*v*) and Rhodamine B in water (15 μL, 0.01% *w*/*v*) were mixed into the fresh FIB-OPEs and SPH-OPEs (1.5 mL) for MCT and OVT staining. An appropriate amount of the stained OPEs was placed on a glass slide, then covered with a coverslip and inverted under the microscope for observation. The images were taken under a CLSM by using the excitation channels at 488 nm and 561 nm.

### 4.11. Droplet Size

The determination of the droplet size of FIB-OPEs and SPH-OPEs was conducted via laser light scattering by an intelligent laser particle sizer (Bettersize 2600, Dandong Bettersize Instruments Ltd., Dandong, China) in the wet mode. Fresh FIB-OPEs and SPH-OPEs obtained in 4.8 were gently injected into the metal cell until the refractive index was in the range of 10–15% to achieve the optimum conditions for the instrument to perform droplet size measurements [18]. Afterward, the injection of FIB-OPEs/SPH-OPEs was stopped, and the measure button was clicked, and the measurement results of the droplet size could be obtained.

### 4.12. Creaming Index

The creaming index of FIB-OPEs and SPH-OPEs was determined as

(1) $$\mathrm{Creaming}\;\mathrm{index}\;(\%)=\frac{H_{c}}{H_{t}}\times 100\%$$

Here, the creaming phase (H_c_) and the total emulsion height (H_t_) were measured by a caliper [56].

### 4.13. Emulsion Stability Evaluation

The physical stability of FIB-OPEs and SPH-OPEs was evaluated by the Turbiscan LAB (Formulaction, Toulouse, France) at room temperature via static multiple-gravity light scattering. In short, 10 mL of the emulsion samples was carefully injected into the specific cylindrical vial, followed by characterization with the Turbiscan instrument at room temperature for 3.5 h; the measurement interval was 25 s. The results obtained, expressed as the Turbiscan Stability Index (TSI), were the function of transmittance (ΔT) and backscattering (ΔBS), which can directly reflect the physical stability of the emulsions.

### 4.14. Rheological Analysis

The rheological properties of FIB-OPEs and SPH-OPEs were performed by an MCR301 rheometer (Anton Paar Co., Ltd., Graz, Austria) in the rotational rheological test mode. The appropriate amount of emulsion samples was added onto the platform using a plastic tip dropper, and the interval between the rotor (PP50) and the platform was set to 1.00 mm. The apparent viscosity of the emulsion samples was measured at 25 ± 0.1 °C with the change of shear rate (0.1–10 s^−1^), and the measurement interval was 2 s. The linear viscoelastic regions of the emulsion samples were obtained using the dynamic strain sweep test at a fixed frequency of 1 Hz at 25 ± 0.1 °C. The frequency sweeps were performed from 1 to 20 Hz, and the storage modulus (G′) and loss modulus (G″) of FIB-OPEs and SPH-OPEs were, respectively, measured at a fixed strain of 0.1%.

### 4.15. Freeze–Thaw Stability

The freeze–thaw stability of FIB-OPEs and SPH-OPEs was evaluated and compared. The freshly prepared OPEs were subjected to three freeze–thaw cycles in total. For one processing cycle, FIB-OPEs and SPH-OPEs were stored in a −18 °C refrigerator for 24 h, and the samples were subsequently taken out and thawed in an oven at 45 °C for 2 h. The freeze–thaw stability of the emulsion samples was evaluated through examining the appearance after freeze–thaw treatments.

### 4.16. Lipolysis and Bioaccessibility of Curcumin Encapsulated in FIB-OPEs and SPH-OPEs

FIB-OPEs and SPH-OPEs loaded with curcumin were first prepared for in vitro gastrointestinal digestion. Briefly, curcumin (5 mg/mL) was dispersed in the mixture of MCT and BW (1.6% *w*/*v*), followed by heating at 80 °C and stirring at 600 rpm until dissolved. Afterward, the vials were removed and cooled to room temperature; then, the curcumin-loaded oleogel was formed. The curcumin-loaded oleogel obtained was used as the oil phase to prepare the OPEs, and the other conditions were the same as those in Section 4.8.

Simulated gastric fluid was formulated via adjusting the pH (pH 2.0) and dissolving sodium chloride (2 mg/mL). The emulsion samples were separately mixed with the obtained simulated gastric fluid (in a 1:4 volume ratio) under constant agitation at 37.0 ± 0.1 °C. The simulated gastric digestion study began when pepsin (1.6 mg/mL) was added. After 120 min, the pH of digesta was raised to 7.5 to inactivate pepsin, thus ending the gastric digestion.

The simulated intestinal digestion required mixing the simulated intestinal fluid (pH 7.5) with an equal volume of the gastric digesta after the simulated gastric digestion. Thereinto, the final concentrations of bile salts, CaCl_2_ and lipase in the mixture were 5 mg/mL, 5 mM and 1.6 mg/mL, respectively. The simulated intestinal digestion was performed under constant agitation at 37.0 ± 0.1 °C for 120 min, during which the pH of the mixture was maintained at 7.5 through injecting NaOH while the additive volume was recorded. After the simulated gastrointestinal digestion, the digesta was centrifuged in a refrigerated centrifuge (4 °C, 10,000× *g*, 40 min). The transparent micellar phase obtained by centrifugation was diluted to an appropriate concentration with absolute ethanol, and the content of curcumin in the micellar phase was determined with an ultraviolet spectrophotometer (UV-2355, Unico, Shanghai, China).

### 4.17. The Light Stability of Curcumin Encapsulated in FIB-OPEs and SPH-OPEs

Fresh MCT-BW oleogel and FIB-OPEs/SPH-OPEs loaded with curcumin obtained in Section 4.16 were exposed to natural light at room temperature. Part of the samples (100 μL) was taken out periodically and thoroughly mixed with absolute ethanol (20.0 mL) to extract residual curcumin. Subsequently, the mixture was centrifuged (11,000× *g*, 30 min), and the centrifugal supernatant was detected at 425 nm using an ultraviolet spectrophotometer (UV-2355, Unico, Shanghai, China) to determine the remaining content of curcumin. The curcumin stability during natural light treatment was reflected by the residual curcumin level, which was calculated using the following equation:

(2) $$\mathrm{Residual}\;\mathrm{level}\;(\%)=\frac{C}{C_{0}}\times 100\%$$

where C was the concentration of curcumin at the specified time point under natural light treatment, and C_0_ was the initial concentration of curcumin.

### 4.18. Statistical Analysis

Each experiment was repeated at least three times. Statistical analyses were performed using Origin 2022 software (Origin Lab Corporation, Northampton, MA, USA). The data obtained were analyzed by the one-way analysis of variance (ANOVA) procedure with Duncan’s multiple comparison test using IBM SPSS Statistics 22.0 (IBM corporation, Chicago, IL, USA) software (*p* < 0.05).

## Figures and Tables

**Table 1:** Gel-sol melting temperatures of beeswax medium-chain triglyceride oil (BW-MCT) oleogels with different addition of BW.

BW Content (% *w*/*v*)	Gel Formation	T_m_ (°C)
1.5	No	-
1.6	Yes	32.5 ± 0.5 ^a^
1.7	Yes	33.0 ± 0.5 ^b^
2.0	Yes	36.0 ± 0.5 ^c^
2.5	Yes	38.5 ± 0.5 ^d^
3.0	Yes	42.0 ± 0.5 ^e^

The different superscript letters in the columns indicate significant differences (*p* < 0.05).

## Data Availability

Data are contained within the article.

## Supplementary Materials

The following supporting information can be downloaded at: https://www.mdpi.com/article/10.3390/gels8080517/s1, Figure S1: Confocal laser scanning micrographs of FIB-OPEs (a–c, corresponding to OVT fibrils, MCT and their overlap, respectively) and SPH-OPEs (d–f, corresponding to OVT spheres, MCT and their overlap, respectively).
