# Supplementary material for: Fibrous and Spherical Aggregates of Ovotransferrin as Stabilizers for Oleogel-Based Pickering Emulsions: Preparation, Characteristics and Curcumin Delivery
Source: Gels. 2022 Aug 19;8(8):517. doi: 10.3390/gels8080517 (PMC9407489; doi:10.3390/gels8080517)
Supplement: Supplementary file 1 [file gels-08-00517-s001.zip › gels-1838287-supplementary.pdf]

gels

Article

Fibrous and Spherical Aggregates of Ovotransferrin as Stabilizers for Oleogel-Based Pickering Emulsions: Preparation, Characteristics and Curcumin Delivery

Qi Zhou [1], Zihao Wei [1,*], Yanan Xu [1] and Changhu Xue [1,2]

[1] College of Food Science and Engineering, Ocean University of China, Qingdao 266003, China
[2] Qingdao National Laboratory for Marine Science and Technology, Qingdao 266235, China
* Correspondence: weizihao@ouc.edu.cn

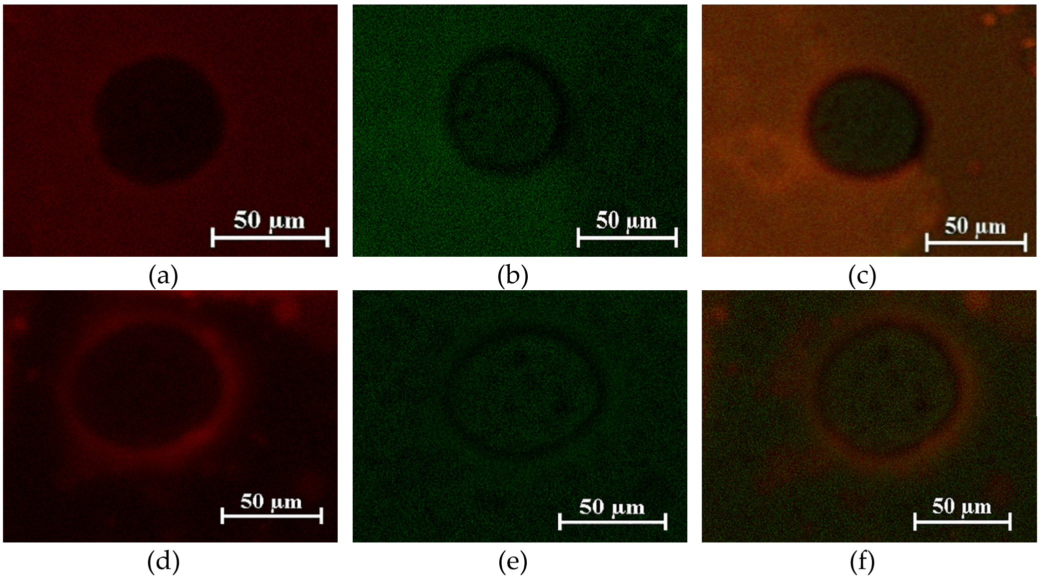

Figure S1. Confocal laser scanning micrographs of FIB-OPEs (a–c, corresponding to OVT fibrils, MCT, and their overlap, respectively) and the SPH-OPEs (d–f, corresponding to OVT spheres, MCT, and their overlap, respectively).
